# The role of endobronchial ultrasound elastography in the diagnosis of mediastinal and hilar lymph nodes

**DOI:** 10.18632/oncotarget.19031

**Published:** 2017-07-06

**Authors:** Ye Gu, Hong Shi, Chunxia Su, Xiaoxia Chen, Shijia Zhang, Wei Li, Fengying Wu, Guanghui Gao, Hao Wang, Haiqing Chu, Caicun Zhou, Fei Zhou, Shengxiang Ren

**Affiliations:** ^1^ Department of Endoscopy, Shanghai Pulmonary Hospital, Tongji University School of Medicine, Shanghai, China; ^2^ Department of Anesthesiology, Shanghai Pulmonary Hospital, Tongji University School of Medicine, Shanghai, China; ^3^ Department of Medical Oncology, Shanghai Pulmonary Hospital, Tongji University School of Medicine & Tongji University School of Medicine Thoracic Cancer Institute, Shanghai, China; ^4^ Department of Respirology, Shanghai Pulmonary Hospital, Tongji University School of Medicine, Shanghai, China

**Keywords:** elastography, EBUS-TBNA, diagnosis, lung cancer, lymph nodes

## Abstract

Endobronchial ultrasound-guided transbronchial needle aspiration (EBUS-TBNA) has been widely used for diagnosis and mediastinal lymph nodes staging in patients with suspicious lung cancer. Ultrasound elastography is a novel sonographical technique that can evaluate tissue compressibility. The aim of the present study was to investigate the diagnostic yield of elastography for differentiating malignant and benign mediastinal lymph nodes. Conventional EBUS B-mode features, including size, shape, border distinction, echogenicity, central hilar structure with central blood vessel and coagulation necrosis were also evaluated. The ultrasonic features were compared with the pathological results from EBUS-TBNA. 133 lymph nodes in 60 patients were assessed. Elastography displayed the highest area under the curve (AUC) (type 3 versus type 1: AUC, 0.825; 95% confidence interval [CI], 0.707-0.910) with an impressive sensitivity (100%) and an acceptable specificity (65%). The combined model covering the four positive criteria (elastography, heterogeneity, size, and shape) showed that the odds ratio for malignance is 9.44 with a 95% CI of 3.99 to 22.32 (*p* <0.0001). The combined model was superior to elastography alone (AUC, 0.851; sensitivity, 89.89%; specificity, 72.73%; *p* <0.0001). This prospective study showed that elastography is a feasible technique for classifying mediastinal lymph nodes, especially in combination with conventional EBUS imaging.

## INTRODUCTION

Lung cancer is one of the most common diagnosed malignant tumors and the leading cause of cancer-related deaths worldwide, with a dismal 5-year survival rate of only 16% [1]. Optimal treatment strategies for patients with this malignance rely on accurate staging and diagnosis. For the majority of patients who are diagnosed at advanced stage, systematic chemotherapy has been the only treatment choice for a long time [2]. In recent years, the presence of epidermal growth factor receptor (*EGFR*) activating mutations and anaplastic lymphoma kinase (*ALK*) chromosomic rearrangements with corresponding tyrosine kinase inhibitors (TKIs) has revolutionized the treatment strategies of patients with advanced non-small cell lung cancer (NSCLC) [3, 4]. It is noteworthy that precise molecular testing for genetic alterations needs adequate specimens. For these inoperable patients, endobronchial ultrasound-guided transbronchial needle aspiration (EBUS-TBNA) has been demonstrated to be a minimally invasive technique for mediastinal node sampling and EBUS-TBNA specimens show high clinical utility for molecular testing, including *EGFR* mutations, *KRAS* mutations and *ALK* rearrangements [5-12].

On the other hand, mediastinal lymph nodes staging is essential for treatment choices for patients without distant metastases. Cervical mediastinoscopy is the “golden standard” for mediastinal nodal staging but this procedure is time-consuming and may cause serious complications [13]. As a minimally invasive technique, EBUS-TBNA has revealed satisfied diagnostic yield for mediastinal nodal staging as compared with mediastinoscopy [14, 15]. However, the diagnostic accuracy of EBUS-TBNA depends on the appropriate selection of lymph nodes, thus, it is still crucial to identify the potentially malignant lymph nodes when performing EUBS-TBNA to reduce unnecessary biopsies. Our previous study has identified several sonographical ultrasonic features that are associated with the malignance of lymph nodes but with varying diagnostic accuracy ranging from 40% to 80% [16], including round shape, distinct margin, heterogeneous echogenicity, presence of coagulation necrosis sign and so on.

Ultrasound elastography is a novel sonographical technique that can evaluate tissue compressibility [17]. In brief, softer tissues deform easier under compression than harder tissues do and neoplastic infiltration may alter the elasticity of tissues and make the infiltrated tissue stiffer. Hence, this technique has potential to distinguish malignant lymph nodes from benign ones. To investigate the diagnostic yield of ultrasound elastography for differentiating malignant and benign mediastinal lymph nodes, we prospectively analyzed the features of ultrasound elastography in 60 patients with 133 lymph nodes who underwent EBUS-TBNA examination in Shanghai Pulmonary Hospital from 1 May 2015 and 31 May 2015.

## RESULTS

### Patients and lymph nodes

A total of 60 patients that were candidates for EBUS-TBNA examination were enrolled in the current study at Shanghai Pulmonary Hospital, Tongji University. In brief, median age of the study population was 62 years (range from 26 to 82), 81.7% (49/60) of patients were male and 31.7% (19/60) of patients were non-smokers.

**Figure 1:**
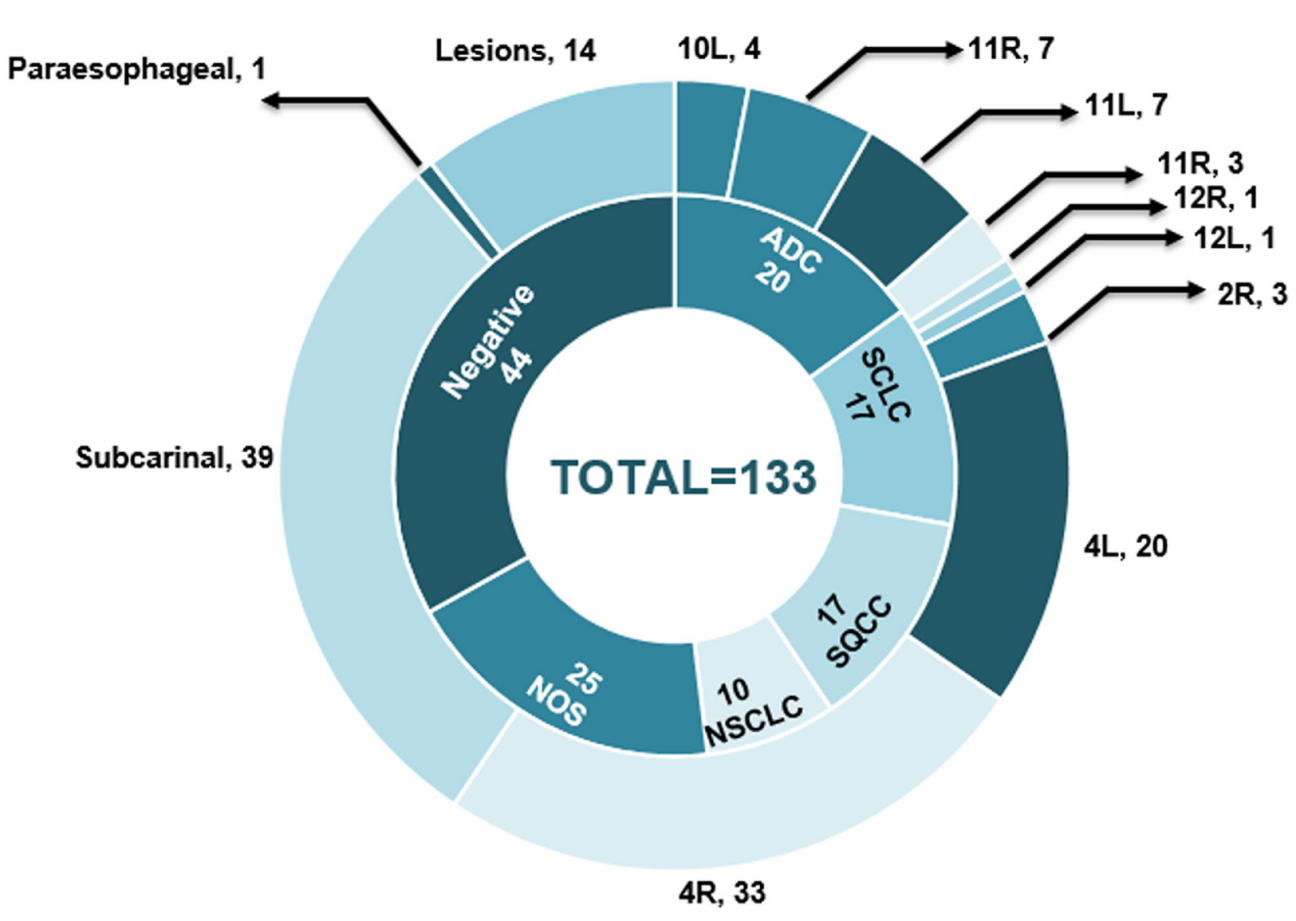
The pathological results and distributions of evaluated lymph nodes ADC, adenocarcinoma; SCLC, small cell carcinoma; SQCC, squamous cell carcinoma; NSCLC, non-small cell lung cancer; NOS, not otherwise specific.

133 lymph nodes in 60 patients were assessed by ultrasound. Among the evaluated lymph nodes, 39 were located in group 7 (subcarinal), 33 were located in group 4R, 20 were located in group 4L, others (41) were located in hilar lymph nodes or lesions. Histologic examinations of the EBUS-TBNA specimens revealed that 66.9% (89/133) of the lymph nodes were malignant (20 were adenocarcinoma, 17 were squamous cell carcinoma, 17 were small cell lung cancer, 10 were NSCLC and 25 were NSCLC-not otherwise specific [NSCLC-NOS]) and 33.1% (44/133) were benign lymph nodes. The pathological results and distributions of evaluated lymph nodes are shown in Figure 1.

### Diagnostic efficacy of EBUS B-mode features and elastography

The diagnostic efficacies of the six EBUS B-mode features and elastography, including the area under the curves (AUCs) with 95% confidence interval (CI), sensitivity, specificity, positive predictive value (PPV), negative predictive value (NPV), and diagnosis accuracy in the diagnosis of malignant lymph nodes are shown in Table 1. Receiver operating characteristic (ROC) analysis shows that elastography, heterogeneity, shape and border margin are positive criteria in the detection of malignant lymph nodes (Table 1).

**Table 1:** Diagnostic efficacy of ultrasound criteria in distinguishing malignant lymph nodes

Lymph node variate	AUC (95%CI)	Sensitivity (95%CI)	Specificity (95%CI)	PPV	NPV	Diagnosis accuracy	*P*
**Short axis ≥10 mm**	0.665 (0.579-0.745)	0.876 (0.808-0.944)	0.455 (0.307-0.602)	0.765	0.645	0.737	0.002
**Round shape**	0.603 (0.515-0.687)	0.798 (0.714-0.881)	0.409 (0.264-0.554)	0.732	0.500	0.669	0.017
**Margin distinct**	0.598 (0.509-0.682)	0.831 (0.754-0.909)	0.364 (0.221-0.506)	0.725	0.516	0.677	0.068
**Echogenicity heterogeneity**	0.711 (0.626-0.786)	0.831 (0.754-0.909)	0.591 (0.446-0.736)	0.804	0.634	0.752	<0.001
**Coagulation necrosis sigh**	0.572 (0.483-0.657)	0.326 (0.228-0.423)	0.818 (0.704-0.932)	0.784	0.375	0.489	0.177
**CHS with central blood vessel**	0.530 (0.442-0.617)	0.281 (0.188-0.374)	0.659 (0.519-0.799)	0.625	0.312	0.406	0.574
**Elastography type**							
**3 versus 1**	0.825 (0.707-0.910)	1.000 (0.916-1.000)	0.650 (0.408-0.846)	0.857	1.000	0.887	<0.001
**2 versus 1**	0.676 (0.565-0.774)	1.000 (0.925-1.000)	0.351 (0.202-0.525)	0.662	1.000	0.714	<0.001

Abbreviations: AUC, area under curve; CI, confidence interval; PPV, positive predictive value; NPV, negative predictive value; CHS, central hilar structure.

Among the investigated ultrasonic criteria, elastography displayed the highest AUC (type 3 *versus* type 1: AUC, 0.825; 95% CI, 0.707-0.910) with an impressive sensitivity (100%) and an acceptable specificity (65%). The PPV, NPV and diagnosis accuracy were 0,857, 1.000 and 0.887, respectively. Consistent with our previous study [16], echogenicity heterogeneity also showed a good diagnostic efficacy in distinguishing malignant lymph nodes, with a moderate AUC of 0.665, a high sensitivity of 0.876 and a moderate specificity of 0.591. Short axis and shape as discriminators showed a diagnosis accuracy of 0.737 and 0.669, indicating a high sensitivity (0.876 and 0.798, respectively) but low specificity (0.455 and 0.409, respectively). Meanwhile, for the variates central hilar structure with central blood vessel (CHS-CBV) and coagulation necrosis, the specificity is low (0.281 and 0.326, respectively), companying with a low diagnosis accuracy (0.406 and 0.489, respectively). Table 2 reveals the correlation between EBUS elastography types and ultrasonic criteria of B-mode features. Notably, when the investigated lymph nodes presented as elastography type 2 or type 3 and any other B-mode features, including short axis > 10mm, round shape, distinct margin and heterogeneous echogenicity, the risk of malignance is over 70% (Table 2).

**Table 2:** Correlation among elastography types, B-mode features of lymph node

	Short axis >10 mm	Round shape	Distinct margin	Heterogeneous echogenicity	Coagulation necrosis sigh	CHS with central blood Vessel
**Elastography Type 1 (*n*=13)**	6/13 (46.2%)	7/13 (53.8%)	11/13 (84.6%)	6/13 (46.2%)	3/13 (23.1%)	3/13 (23.1%)
**Elastography Type 2 (*n*=71)**	56/71 (78.9%)	48/71(71.8%)	51/71 (71.8%)	50/71 (70.4%)	15/71 (21.1%)	23/71 (32.4%)
**Elastography Type 3 (*n*=49)**	40/49 (85.7%)	42/49 (85.7%)	40/49 (81.6%)	36/49 (73.5%)	19/49 (38.8%)	14/49 (28.6%)

**Figure 2:**
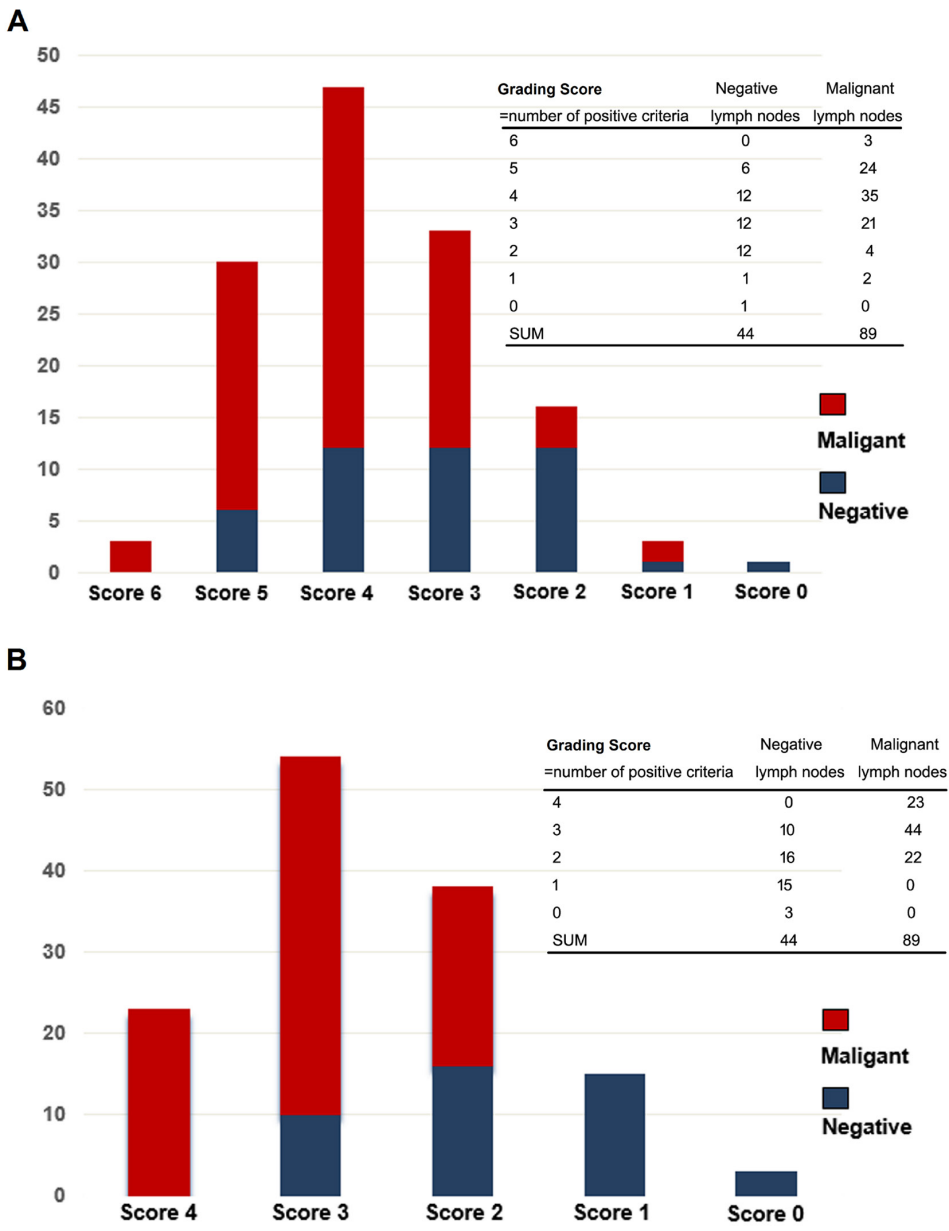
The diagnosis efficacy of the combined model for differentiating malignant and benign mediastinal lymph nodes **A.** A combined model including the current six conventional EBUS B-mode features (size, shape, border distinction, echogenicity, central hilar structure with central blood vessel and coagulation necrosis). **B.** A combined model only including positive ultrasonic criteria (elastography, heterogeneity, size, and shape). EBUS, endobronchial ultrasound.

**Figure 3:**
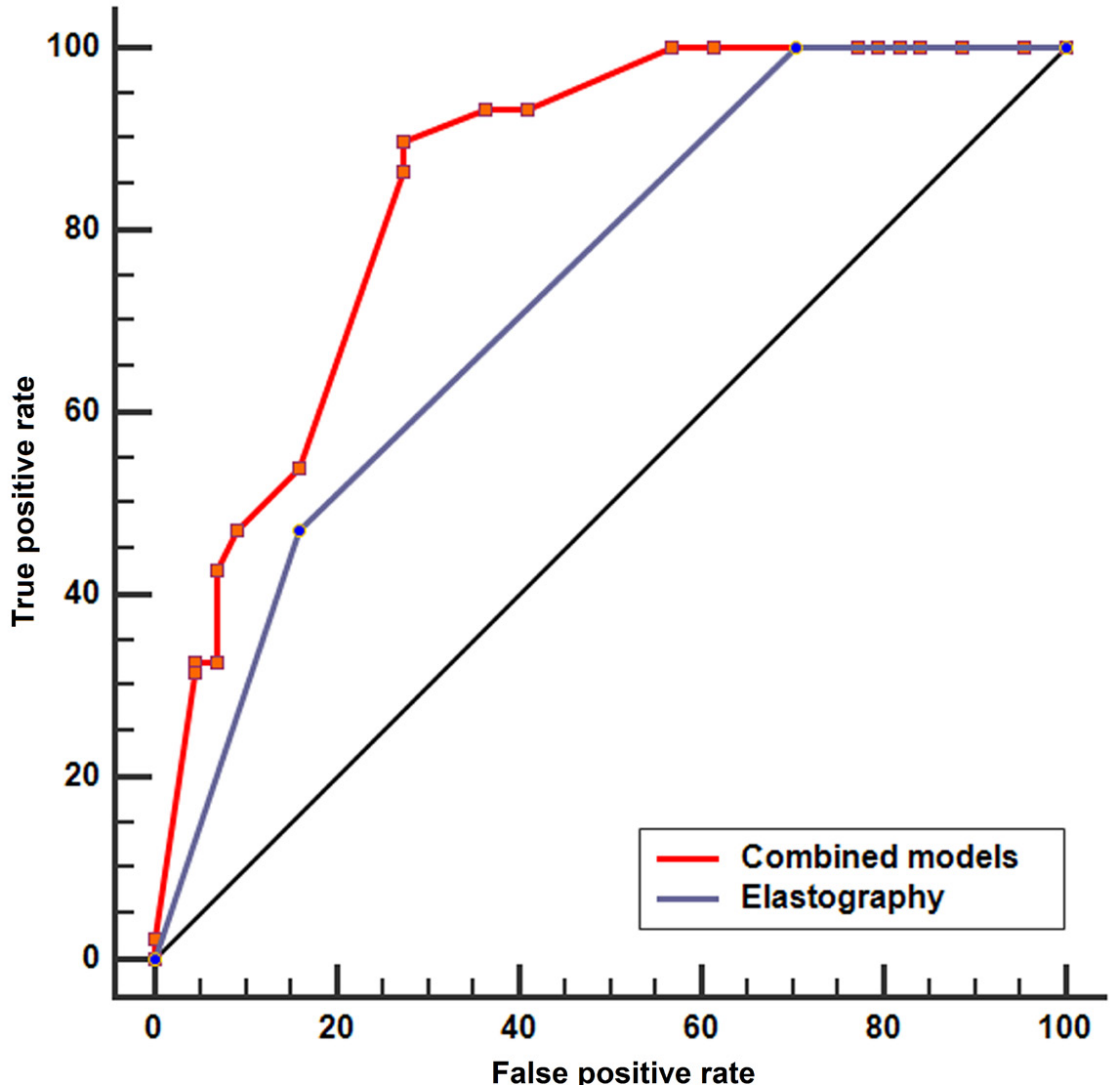
The ROC analysis of the combined model covering the four positive criteria and elastography alone ROC analysis demonstrated that the combined model covering the four positive criteria is superior to elastography alone (AUC, 0.851; sensitivity, 89.89%; specificity, 72.73%; *p* < 0.0001). ROC, receiver operating characteristic; AUC, area under the curve.

In order to improve the diagnostic efficacy of positive criteria in the detection of malignant lymph nodes, we investigated a model of grading scores where the number of positive criteria is counted (elastography, heterogeneity, size, and shape) (Figure 2). Figure 2A shows number of patients with accordant number of the current six conventional EBUS B-mode features. When indicating grading score of 3-6 as “high risk” and 1-2 as “low risk”, the odds ratio (OR) for malignance is 5.99 with a 95% CI of 2.09 to 17.19 (*p* < 0.0001). Figure 2B shows number of patients with accordant number of only positive ultrasonic criteria including elastography. Similarly, when indicating grading score of 3-4 as “high risk” and 1-2 as “low risk”, the OR for malignance is 9.44 with a 95% CI of 3.99 to 22.32 (*p* < 0.0001), suggesting that the presence of more than two positive criteria indicates malignancy of a lymph node and the model only including positive criteria is simple and adequate in distinguishing malignant lymph nodes. Further ROC analysis demonstrated that the combined model covering the four positive criteria is superior to elastography alone (AUC, 0.851; sensitivity, 89.89%; specificity, 72.73%; *p* < 0.0001) (Figure 3).

## DISCUSSION

At present, National Comprehensive Cancer Network (NCCN) suggests mediastinoscopy or EBUS-TBNA as recommended procedures for the evaluation of mediastinal lymph nodes for patients with lung cancer who are candidates for surgery [18]. Additionally, EBUS-TBNA is also presented as a minimally invasive technique for the accurate diagnosis of patients with lung cancer [14, 15]. Although EBUS-TBNA reveals a high specificity and PPV for the diagnosis of malignant lymph nodes [14], the diagnostic accuracy of this procedure depends on the accurate selection of targeted lymph nodes. Conventionally, the selection of target lesions during EBUS-TBNA is based on insensitive size and morphologic criteria [16], such as size, shape, border distinction, echogenicity, central hilar structure and coagulation necrosis, thus, lacking desired accuracy for differentiating malignant and benign lymph nodes. PET shows promising clinical utility but still has some limitations for classifying small nodal metastases [19]. In contrast, ultrasound elastography, designed to evaluate the stiffness of target tissues, has been established as a novel sonographical technique for the characterizing lymph nodes. Previous meta-analysis has demonstrated that EBUS elastography display a sensitivity of 88% and a specificity of 85% in differentiating malignant and benign lymph nodes [20]. To date, EBUS elastography has been proved to be a useful tool for the diagnosis and assessment of breast cancer [21], thyroid nodules [22], prostate cancer [23], and esophagogastric cancer staging et al. [24].

Interestingly, previous studies also evaluated the clinical utility of EBUS elastography for the diagnosis of mediastinal and hilar lymph nodes [25-28]. In the study by Izumo et al. [25], qualitative elastographic pattern analysis showed that in 75 evaluated lymph nodes, the sensitivity, specificity, PPV, NPV and diagnostic accuracy rates were 100%, 92.3%, 94.6%, 100% and 96.7%, respectively for malignance prediction. A recent study by Rozman et al. assessed the diagnostic value of elastography strain ratio of mediastinal lymph nodes in patients with suspicion of lung cancer [26]. In 32 patients with 80 suspicious mediastinal lymph nodes, the diagnostic accuracy, sensitivity, specificity, PPV and NPV for differentiating malignant and benign lymph nodes were 86.25%, 88.24%, 84.78%, 81.08%, and 90.70%, respectively. Consistent with the findings from above studies, our study including 60 patients with 133 evaluated lymph nodes, the largest sample size to our knowledge, also demonstrated the significantly clinical utility of EBUS elastography for classifying suspicious mediastinal lymph nodes. Our study proved EBUS elastography as a non-invasive and reliable method that adds complementary information to current conventional EBUS imaging and help differentiating benign and malignant lymph nodes.

The high diagnostic accuracy (88.7%), PPV (85.7%), and NPV (100%) of EBUS elastography in our study has two aspects of clinical implications. For patients with lung cancer who are candidates for surgery, if the evaluated lymph nodes imaging present as elastography Type 3, EBUS-TBNA should be performed even the short axis is less than 10mm or presenting with other benign signs. In case of negative-TBNA or in situations where the puncture is not available, cautions should be taken when patients with Type 3 lymph nodes and other methods such as PET should be used to help characterizing the evaluated lymph nodes. On the other hand, for patients with inoperable and advanced lung cancer, duo to the high NPV, EBUS-TBNA should be performed to in Type 3 lymph nodes to reduce unnecessary damage and shorten the duration of procedures. Additionally, elastography can also help to guide the puncture area in a non-necrotic part of the suspicious lymph nodes (blue area) to improve diagnostic accuracy and specimen quality for diagnosis or further molecular testing.

In the present study, we also evaluated the diagnostic value of EBUS B-mode features for mediastinal lymph nodes as compared with elastography. In concordance with our previous study [16], heterogeneity, size and shape also showed a relatively good diagnostic accuracy ( > 60%) for the diagnosis of lymph nodes, except for CHS-CBV and coagulation necrosis. Although the specificity in our study is lower than that in previous studies [25-28] (Supplementary Table 1), the PPV is comparable. Furthermore, the sensitivity and NPV is 100% in our study, which is higher than previous studies except for the study by Izumo et al [25]. In order to improve the diagnostic efficacy, we introduced a grading score model of the four positive criteria to assess lymph nodes. In this model, if the evaluated lymph nodes met all the four positive criteria, all of the 23 lymph nodes were malignant. The model suggested that the presence of more than two positive criteria indicates an OR for malignancy is as high as 9.44 and the model only including the four positive criteria is simple and adequate in distinguishing malignant lymph nodes. Further ROC analysis of the combined model can improve the specificity from 65% to 72.7%.

Several limitations should be mentioned in the present study. First, the sample size was still limited and relatively small. Second, previous studies suggested that strain ratio of elastography may be an objective indicator for the classification of lymph nodes [26, 27]. The diagnostic value of strain ratio needs to be evaluated in our center. However, the strain ratio was determined from “frozen” EBUS images, selection bias cannot be ignored when selecting reference area. The dynamic evaluation of region of interest may have some advantages. Finally, although the elastography data was collected prospectively and the movies of elastography and pathological results were blinded to raters in our study, prospective and multi-center trials are needed to confirm the findings in the future.

In conclusion, this prospective study showed a significantly clinical utility of elastography for classifying suspicious mediastinal lymph nodes with a high PPV, NPV and diagnostic accuracy. Our study also showed that the diagnostic yield will be improved when combined EBUS elastography with the current conventional EBUS imaging, such as heterogeneity, size, and shape.

## MATERIALS AND METHODS

### Patient selection

This single-center study prospectively enrolled consecutive patients with 5-mm slice, single contrast injection chest computed tomography (CT) who had mediastinal lymphadenopathy between 1 May 2015 and 31 May 5 2015 at Shanghai Pulmonary Hospital. Exclusive criteria included distant metastases, severe co-morbidities unable to tolerate surgical procedures (severe coronary heart disease, uncontrolled hypertension or malignant arrhythmia et al.) and mediastinal tumor infiltration. This study was approved by the Ethics Committee of Shanghai Pulmonary Hospital, Tongji University and all patients who participated in this study signed an informed consent before any study related procedure.

### EBUS procedure

All examinations were carried out by the same experienced bronchoscopist (Y.G). EBUS procedures were performed with a convex probe EBUS (BF-UC260FOL8; Olympus, Tokyo, Japan, 7.5 MHz) and the ultrasound processor EU-C2000 (Olympus). After local anesthesia to the pharynx with 4% lidocaine spray (10ml), the convex probe EBUS was inserted through the nasal cavity or oral route, with intermittent instillation of 2% lidocaine (2-4ml per time).

**Figure 4:**
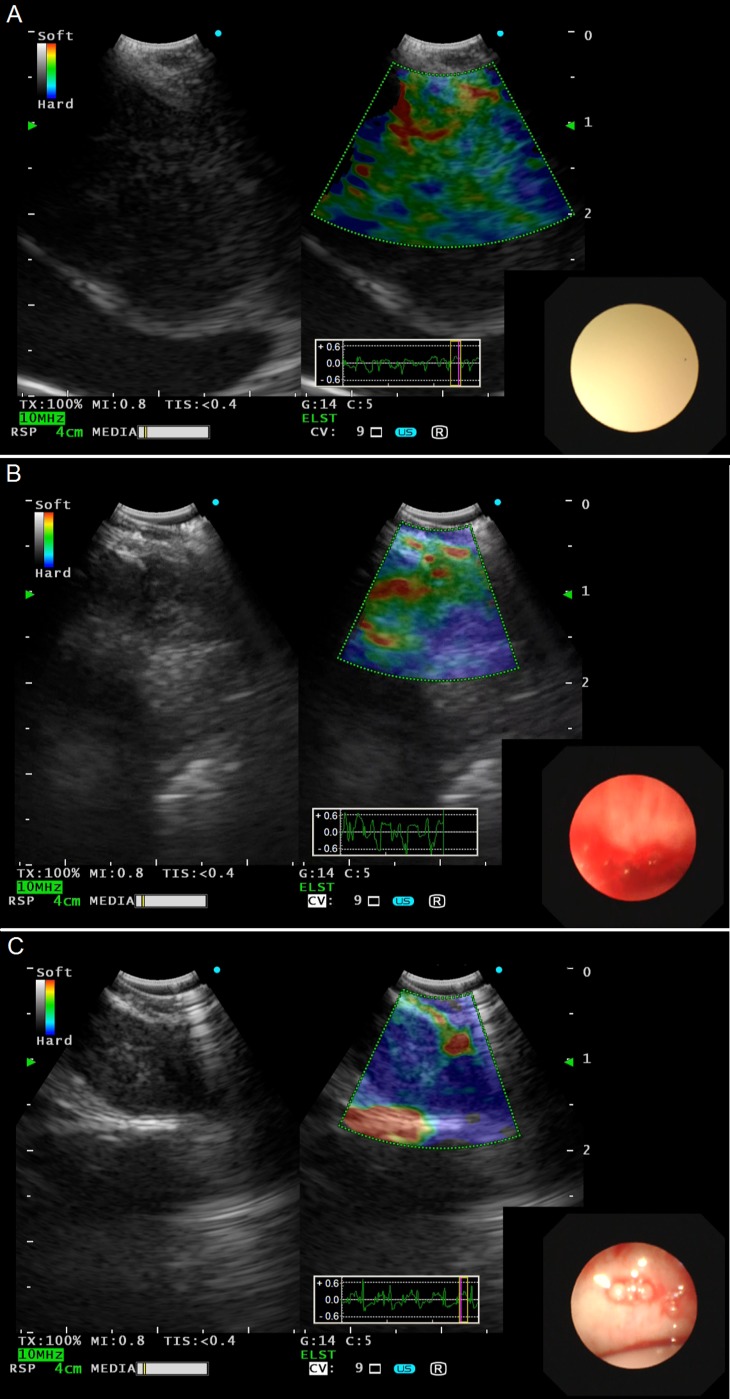
Representative lymph nodes on EBUS elastography **A.** Type 1: dominant colors were green, yellow or red. **B.** Type 2: part blue, part green, yellow or red. **C.** Type 3: dominant color was blue. EBUS, endobronchial ultrasound.

Real-time EBUS B-mode and elastography were recorded as digital movies prospectively. Strain elastography was used to evaluate lymph nodes in our study. Ultrasonic criteria of EBUS B-mode and vascular pattern on power Doppler included size, shape, border distinction, echogenicity, CHS-CBV and coagulation necrosis, sighs that have been previously demonstrated to predict malignancy or benign mediastinal lymph nodes [16]. After recording the movies of EBUS B-mode ultrasound criteria, the procedure was switched to strain elastography mode and all lymph nodes that were candidates for EBUS-TBNA were evaluated by strain elastography. The elastography image was generated by vascular pulsations and respiratory movement as the result of tissue compression. After comparing the scanned area with the surrounding normal tissue, elastography images were reconstructed and translated into a color signal that was overlaid on the B-mode image. Blue was indicated to represent hard tissue; otherwise, green and yellow/red were indicated to represent mediate, soft tissue, respectively. Based on the dominant colors and their distributions in the target lymph nodes, elastographic patterns were classified into 3 types as previously described and shown in Figure 4 [25]: Type 1 (Figure 4A), dominant colors were green, yellow or red; Type 2 (Figure 4B), part blue, part green, yellow or red; Type 3 (Figure 4C), dominant color was blue.

After non-invasive evaluation, EBUS-TBNA was performed using a 22-gauge needle (NA-201SX-4022; Olympus). The puncture with negative pressure was performed for about 10-20 passes a time, and 3 aspirations per lymph node under the guidance of real-time EBUS. The aspirated material was smeared onto glass slides, afterwards were air-dried for immediate evaluation by an on-site cytopathologist to confirm adequate cell materials. Additional aspirated materials were collected in liquid formalin for further pathological evaluation or molecular testing. The cytological analysis was afterwards performed by an experienced pathologist who was blinded to EBUS B-mode features and elastography types.

Two raters (F.Z, S.R) evaluated independently the blinded movies of EBUS B-mode features and elastography (baseline characteristics and final pathological results of evaluated patients were blinded to the two raters). Any disagreements were resolved by consensus.

### Statistical analysis

ROC analysis was performed to compare the specificity/sensitivity of EBUS B-mode features and elastography with pathological results of EBUS-TBNA in distinguishing malignant lymph nodes. The AUCs were calculated. Sensitivity, specificity, accuracy, PPVs, and NPVs of ultrasonic criteria of EBUS B-mode and elastography were calculated. Binary logistic regression analysis was used to determine whether elastography could improve the diagnostic accuracy when in combination with the conventional EBUS B-mode features. Statistical analysis was performed using SPSS software (18.0, SPSS Inc., Chicago, IL) and MedCalc (version 13.0.0). All *p* values were based on two-sided testing, where *p* values less than 0.05 were considered significant.

## SUPPLEMENTARY MATERIALS TABLE


