# Supplementary material for: The role of endobronchial ultrasound elastography in the diagnosis of mediastinal and hilar lymph nodes
Source: Oncotarget. 2017 Jul 6;8(51):89194–202. doi: 10.18632/oncotarget.19031 (PMC5687681; doi:10.18632/oncotarget.19031)
Supplement: Supplementary file 1 [file oncotarget-08-89194-s001.pdf]

The role of endobronchial ultrasound elastography in the diagnosis of mediastinal and hilar lymph nodes

SUPPLEMENTARY MATERIAL

Supplementary Table 1: Summary studies of diagnostic yield of elastography in differentiating mediastinal lymph nodes.

Study	LN	Sensitivity	Specificity	PPV	NPV	Diagnosis accuracy
**Okasha H, 2013**	84	76.3%	100%	100%	84.7%	NR
**Izumo T, 2014**	75	100%	92.3%	94.6%	100%	96.7%
**He HY, 2015**	68	88.1%	80.8%	88.1%	80.8%	85.3%
**Rozman A, 2015**	80	88.2%	84.7%	81.0%	90.7%	86.2%
**Gu Y.**	133	100%	65%	85.7%	100%	88.7%

Abbreviations: LN, lymph node; PPV, positive predictive value; NPV, negative predictive value.
